# AI-driven multi-omics integration in breast cancer: clinical applications, immunotherapy prediction, and translational challenges

**DOI:** 10.3389/fmolb.2026.1875788

**Published:** 2026-09-24

**Authors:** Zi-Yao Wang, Na Liu, Min-Bin Chen

**Affiliations:** Department of Radiotherapy and Oncology, Affiliated Kunshan Hospital of Jiangsu University, Kunshan, China

**Keywords:** artificial intelligence, breast cancer, multimodal data, multi-omics, precision medicine, prognosis

## Abstract

Breast cancer remains a major global threat to women’s health, and its marked molecular, spatial, and temporal heterogeneity continues to limit the accuracy of early diagnosis, risk stratification, and treatment-response prediction. Conventional single-dimensional diagnostic and therapeutic models are often insufficient to capture the dynamic complexity of the tumor microenvironment (TME). In this context, artificial intelligence (AI) provides powerful tools for high-dimensional feature extraction, multimodal data integration, and cross-scale modeling of heterogeneous biological and clinical information. AI-enabled approaches can link microscopic molecular alterations with macroscopic imaging and pathological phenotypes, while also characterizing complex cellular interactions within the TME. This review summarizes current applications of AI-driven multimodal and multi-omics integration in breast cancer, focusing on early detection, precision diagnosis, immunotherapy-response prediction, drug-sensitivity assessment, and prognostic evaluation. We also discuss key translational challenges, including data heterogeneity, batch effects, interpretability, privacy protection, regulatory considerations, and clinical workflow integration. Overall, AI-driven multi-omics strategies offer a promising framework for improving individualized treatment selection and real-time monitoring in breast cancer, although robust prospective validation and multidisciplinary implementation are still required before routine clinical adoption. Compared with previous modality-specific reviews, this article emphasizes cross-modal evidence appraisal, clinical-maturity stratification, data-quality constraints, foundation model opportunities, and regulatory requirements for translating AI-enabled multi-omics from exploratory research to clinically auditable decision support.

## 1 Introduction

Breast cancer is one of the most important public health challenges affecting women worldwide (Bray et al., 2024). Globally, breast cancer accounts for approximately one-third of all female malignancies, with a mortality rate representing about 15% of the total diagnosed cases. The global distribution of breast cancer is influenced by a complex interplay of genetic, environmental, and lifestyle factors (Siegel et al., 2024; Xu et al., 2023). In many countries, it also remains one of the leading causes of cancer-related death among women (Sung et al., 2021). Although screening programs and improvements in surgery, endocrine therapy, and supportive care have improved overall survival (Giaquinto et al., 2024), prevention and disease control remain challenging in the context of population aging and changing lifestyles (GBD, 2023 Breast Cancer Collaborators, 2026). For patients at high risk of recurrence or metastasis, traditional single-dimensional treatment models have reached important efficacy limits, underscoring the need for more precise and individualized strategies (Ran D. et al., 2025).

Breast cancer is not a single disease but a heterogeneous group of malignancies characterized by substantial spatial and temporal heterogeneity at the molecular level (Barzaman et al., 2020). This marked intratumoral heterogeneity (ITH) represents a major barrier to current clinical diagnosis and treatment (An et al., 2024). At the diagnostic level, conventional pathological classification based on ER, PR, HER2, and Ki-67 is insufficient to fully capture tumor aggressiveness and invasive potential (Intriago-Baldeón et al., 2025). As a result, some early-stage patients may receive overtreatment, whereas high-risk patients with occult micrometastases may miss the optimal window for intervention (Prakash et al., 2024; Ni et al., 2025). Such heterogeneity directly affects the efficacy of chemotherapy and immunotherapy (Taylor et al., 2024) and is significantly associated with 5-year survival (Chen H. et al., 2025).

In the rapidly evolving field of cancer immunotherapy, treatment response varies substantially among patients, particularly in triple-negative breast cancer (TNBC) (Yang and Wang, 2025). Because of the complexity of the tumor immune microenvironment (TIME), a single biomarker—such as PD-L1 expression or tumor mutational burden (TMB)—is often insufficient to accurately identify patients who are likely to benefit from therapy (Yin et al., 2024) and may fail to anticipate immune escape or severe immune-related adverse events (irAEs) (Bianchini et al., 2022; Tong et al., 2025). Traditional binary classification methods do not adequately account for interactions among tumor metabolism, stromal architecture, genomic instability, and host immunity (Wik et al., 2025). Therefore, the mechanisms of immune evolution in breast cancer need to be re-examined from a systems-biology perspective (Chai et al., 2025).

With rapid advances in high-throughput sequencing, single-cell RNA sequencing (scRNA-seq), spatial transcriptomics, and radiomics, cancer research has entered an era of large-scale omics data (Dezem et al., 2024). However, single-omics technologies are inherently limited by their observational dimensions and may generate information silos, making it difficult to reconstruct the complex networks linking genes, metabolism, the microenvironment, and host immunity (Zhao et al., 2026). For multidimensional, large-scale, and highly nonlinear biological datasets, traditional statistical methods are often insufficient, whereas artificial intelligence (AI) offers powerful capabilities for high-dimensional feature extraction and cross-modal integration (Hsu et al., 2026). Figure 1 outlines the evolution from conventional single-dimensional approaches to AI-driven multi-omics integration.

**FIGURE 1:**
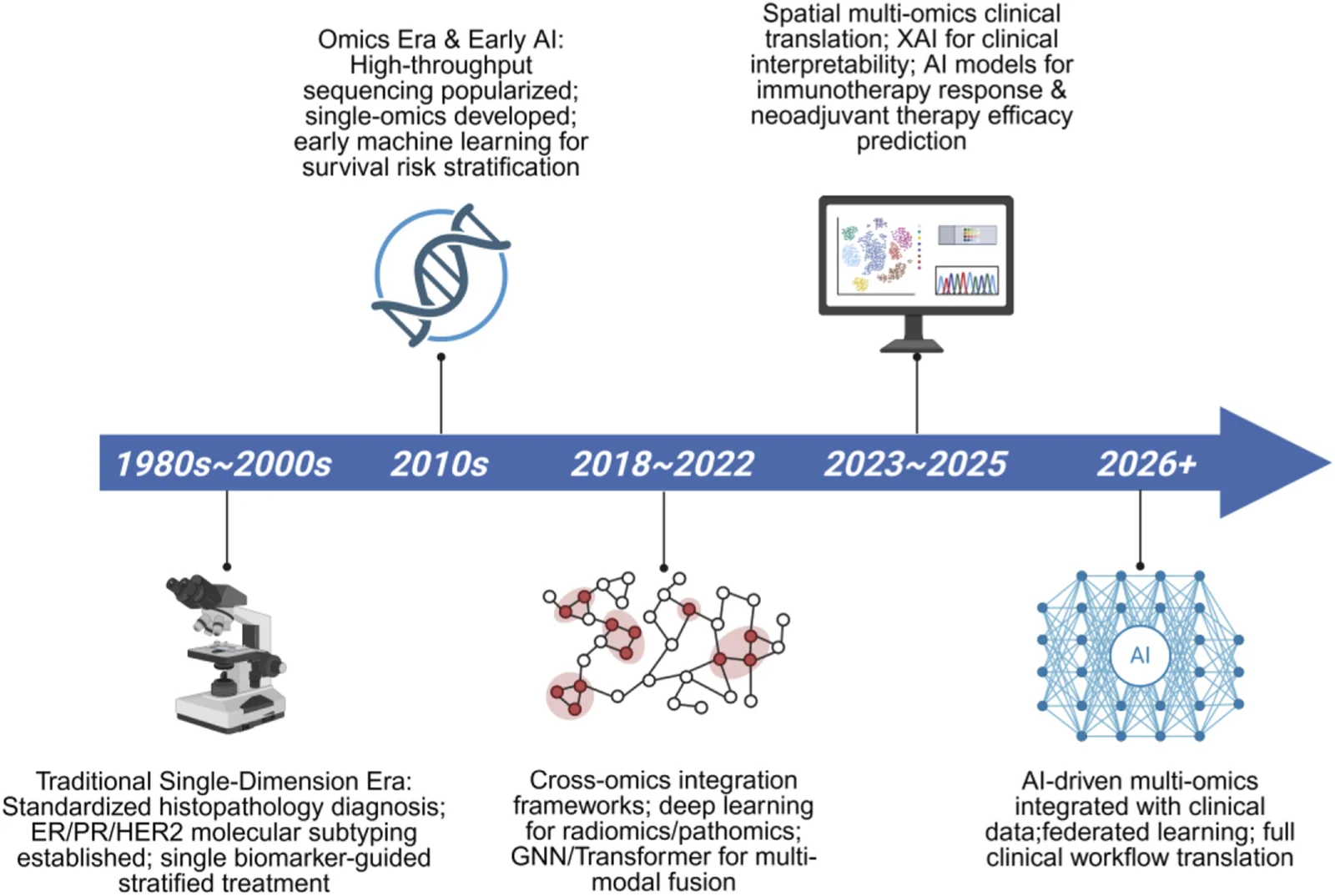
Timeline of Omics Development. The figure outlines the evolution of artificial intelligence in clinical practice over the past half-century, from traditional single-omics approaches to the integration of multi-omics with artificial intelligence.

AI applications in oncology depend strongly on the type of data analyzed: for structured clinical variables, classical machine-learning methods are commonly used for survival prediction and risk stratification (Javanmard et al., 2025); for pathological and radiological images, convolutional neural networks (CNNs) can extract spatial features for lesion segmentation (Perez-Lopez et al., 2024; Islam et al., 2024); and for sequence data such as genomes or medical records, Transformers and recurrent neural networks (RNNs) can model long-range dependencies and support biomarker discovery. Large language models (LLMs) may further accelerate knowledge extraction and validation (Truhn et al., 2024; Fountzilas et al., 2025). Through multimodal fusion, AI can reduce barriers among omics layers, map microscopic genotypes to macroscopic imaging phenotypes, and identify latent biomarker combinations (Acosta et al., 2022). By integrating genomics, transcriptomics, proteomics, pathology, and imaging, this strategy enables comprehensive characterization of tumor mutations, gene expression, microenvironmental composition, and spatial invasion patterns (Jiang et al., 2026; Datwani et al., 2025), thereby supporting more accurate prediction of biological behavior, prognostic risk assessment, treatment optimization, and the development of innovative therapeutic strategies (Wu and Li, 2025).

Existing articles have addressed AI-enabled multi-omics integration in pan-cancer research, neoadjuvant chemotherapy prediction, digital pathology, radiomics, multimodal data fusion, foundation models, and breast-cancer screening (Liu F. et al., 2026). However, most prior work is either modality-specific, task-specific, or pan-cancer in scope. The present review complements these prior efforts by taking a breast-cancer-specific and clinically oriented evidence-appraisal perspective, with particular emphasis on evidence quality, external validation, clinical maturity, regulatory readiness, and translational feasibility across radiomics, pathomics, genomics, transcriptomics, proteomics, metabolomics, liquid biopsy, single-cell sequencing, and spatial transcriptomics (Freeman et al., 2021). Table 1 compares the scope of recent reviews and highlights the specific contributions of the present review.

**TABLE 1:** Comparison with recent reviews and specific contribution of the present review.

Prior review focus	Main contribution	Specific contribution of the present review	References
AI for multi-omics and clinical-data integration in cancer research	Reviews the application of AI for integrating genomics, transcriptomics, proteomics, imaging, pathology, and clinical records to support early diagnosis, patient stratification, treatment-response prediction, and drug-resistance analysis	This prior work provides a broad pan-cancer framework. The present review adopts a disease-specific perspective by focusing on breast cancer and relating AI-enabled multi-omics integration to clinically relevant endpoints, including molecular subtype classification, neoadjuvant treatment response, immunotherapy prediction, drug sensitivity, prognosis, and translational feasibility	(Liu et al., 2026a)
AI-integrated multi-omics and multimodal studies for neoadjuvant chemotherapy in breast cancer	Summarizes AI-driven integration of single-omics, multimodal, and multi-omics data for predicting neoadjuvant chemotherapy response, identifying biomarkers, characterizing tumor heterogeneity, and profiling the immune microenvironment	This literature primarily focuses on the neoadjuvant chemotherapy setting. The present review places this topic within a broader clinical framework that also includes early diagnosis, immunotherapy-response prediction, drug sensitivity, prognosis, clinical maturity, data governance, and regulatory considerations	(Ye et al., 2026)
Digital pathology AI and clinical oncology implementation	Critically examines the development and implementation of AI-driven digital pathology, with emphasis on validation datasets, regulatory approval, reimbursement, workflow integration, and generalizability	This literature mainly addresses pathology AI as a clinical and technical domain. The present review considers pathomics as one component of breast-cancer multi-omics integration and discusses its connections with radiomics, genomics, transcriptomics, proteomics, metabolomics, liquid biopsy, and spatial omics	(Ye et al., 2026)
Large language models and multimodal foundation models for precision oncology	Discusses large language models and multimodal foundation models in oncology research, including applications involving clinical text, imaging, pathology, molecular data, trial matching, and precision-oncology decision support	These discussions are generally not specific to breast cancer. The present review incorporates LLMs and foundation models into the context of breast-cancer multi-omics integration and further considers retrieval-augmented systems, hallucination risk, model auditability, regulatory monitoring, and clinical-readiness constraints	(Truhn et al., 2024)
Radiomics translation into clinically useful tests	Outlines key criteria for translating radiomics into clinically useful tests, including standardization, validation, reproducibility, benefit?risk assessment, and clinical utility	This work is primarily radiomics-focused. The present review uses these translational criteria to evaluate radiomics as one layer of AI-enabled breast-cancer multi-omics integration and to compare its relative clinical maturity with that of emerging omics technologies	(Huang et al., 2023)
Deep-learning-based multimodal data fusion for cancer biomarker discovery	Reviews early, intermediate, and late fusion strategies, representation learning, data sparsity, model interpretability, and biomarker discovery using multimodal cancer data	This literature is mainly method-oriented and pan-cancer in scope. The present review links fusion architectures to breast-cancer-specific clinical tasks and evaluates their relevance in relation to study quality, overfitting, external validation, reproducibility, and translational feasibility	(Steyaert et al., 2023)

## 2 Methods: literature search and selection strategy

This study was conducted as a structured narrative review of artificial intelligence (AI)-enabled multi-omics and multimodal research in breast cancer. PubMed, Web of Science, Scopus, Embase, IEEE Xplore, and Google Scholar were searched for relevant English-language publications available up to April 2026. The search combined terms related to breast cancer, AI methods, data modalities, clinical applications, and clinical translation. Representative search terms included “breast cancer,” “artificial intelligence,” “machine learning,” “deep learning,” “multi-omics,” “multimodal data,” “radiomics,” “pathomics,” “genomics,” “transcriptomics,” “proteomics,” “metabolomics,” “liquid biopsy,” “single-cell sequencing,” “spatial transcriptomics,” “immunotherapy,” “neoadjuvant therapy,” “pathological complete response,” “drug sensitivity,” “prognosis,” “external validation,” and “clinical translation.”

Original research articles were considered when they examined AI-based analysis or integration of molecular, imaging, pathological, spatial, or clinical data in relation to breast cancer diagnosis, molecular classification, treatment-response prediction, immunotherapy stratification, drug sensitivity, prognosis, or clinical implementation. Relevant reviews and methodological studies were used to provide context regarding multimodal integration, model validation, reproducibility, and interpretability. Reporting guidelines and regulatory documents were also considered when discussing evidence quality, governance, and clinical implementation. Publications were excluded if they were unrelated to breast cancer, focused exclusively on non-oncological applications, lacked sufficient methodological information for interpretation, or did not address a clinically or biologically relevant application of AI-enabled omics or multimodal analysis.

Because this was a narrative rather than a systematic review, studies were selected for their relevance to the scope and clinical questions of the article rather than through a formal meta-analytic selection framework. Greater emphasis was placed on studies with independent external validation, multicenter cohorts, clearly defined and clinically meaningful endpoints, or clear translational relevance. Exploratory and proof-of-concept studies were also included when they represented important developments in emerging areas, particularly single-cell sequencing, spatial transcriptomics, and metabolomics; however, these studies were interpreted separately from evidence supported by external, multicenter, or prospective validation.

During narrative synthesis, the maturity of the evidence was considered in relation to study design, cohort size, validation setting, endpoint relevance, reproducibility, and proximity to prospective clinical implementation. Retrospective, single-center, or internally validated studies were not interpreted as providing the same level of evidence as multicenter, externally validated, or prospective investigations. Where reported, attention was also paid to potential overfitting, population bias, model calibration, and the transparency of model development and validation.

Because the included studies differed substantially in data modality, patient population, clinical endpoint, AI architecture, and validation strategy, a quantitative meta-analysis and direct comparison of reported performance metrics were not considered appropriate. The evidence was therefore synthesized narratively, with particular attention to methodological quality, validation status, potential bias, generalizability, clinical utility, translational maturity, and barriers to implementation.

## 3 Fundamentals of AI-driven multi-omics integration strategies and algorithms

The core of multi-omics research is integration. The effective combination of structured genetic data with unstructured imaging data depends on appropriate AI model design (Waqas et al., 2024a). Before data are entered into AI models, information from different sources requires rigorous preprocessing and feature engineering to reduce technical noise and extract clinically meaningful features (Perez-Lopez et al., 2024).

Batch-effect correction reduces non-biological variation generated across sequencing platforms, laboratories, and experimental batches (Perez-Lopez et al., 2024). Although ComBat is widely used, its performance may be limited when data from different cohorts, such as TCGA and METABRIC, are integrated (Fountzilas et al., 2025). Emerging AI approaches, including ComBat-seq for RNA-seq count data and generative adversarial network (GAN)-based denoising algorithms, are increasingly used to preserve biological signals more effectively (Hsu et al., 2026). Tools such as SPIRAL combine GraphSAGE with Wasserstein-distance domain adaptation to align feature distributions across batches. Missing-value imputation is another key step because multi-omics datasets often contain incomplete layers, such as absent proteomic profiles for some patients. Deep generative models, including MIWAE, and Transformer-based imputation methods can infer missing values from other omics layers and often outperform simple mean imputation (Hsu et al., 2026; Ran R. et al., 2025). Beyond batch effects and missing-value imputation, broader data-quality challenges are critical determinants of the validity of AI-enabled multi-omics models (Collins et al., 2024). Inter-pathologist variability may affect diagnostic labels, tumor-region delineation, tumor-infiltrating lymphocyte assessment, and annotation masks used for pathomics model training (Garcia et al., 2022). Incomplete clinical metadata, including treatment sequence, dose intensity, recurrence status, follow-up duration, and subsequent lines of therapy, may introduce unmeasured confounding and compromise cross-cohort comparability (Andaur Navarro et al., 2021). Inconsistent definitions of clinically meaningful endpoints, such as pathological complete response, progression-free survival, disease-free survival, and overall survival, further hinder the interpretation and benchmarking of model performance across studies (Tolaney et al., 2021). Technical heterogeneity also extends well beyond sequencing batch effects. Differences in sequencing chemistry, library preparation, tissue fixation, slide preparation, staining protocols, scanner calibration, imaging acquisition protocols, reconstruction parameters, segmentation procedures, and radiomics feature-extraction pipelines can substantially alter model inputs (Zwanenburg et al., 2020). If these factors are not systematically documented, harmonized, and controlled, AI models may capture institution-specific artifacts rather than reproducible disease biology. Therefore, standardized metadata collection, predefined quality-control workflows, preregistered clinical endpoints, transparent reporting of missingness, and detailed documentation of acquisition and preprocessing parameters should be regarded as minimum requirements for robust, reproducible, and clinically translatableAI-driven multi-omics research (Sounderajah et al., 2025).

### 3.1 Multi-omics integration strategies: from early fusion to deep interaction

According to the stage at which data fusion occurs, AI architectures can be broadly divided into three strategies with distinct applications in breast cancer research. The first is early fusion, also known as concatenation (Waqas et al., 2024a). This approach directly concatenates feature vectors from different omics layers into a high-dimensional input vector. For example, genomic, transcriptomic, pathological, and clinical features can be combined after feature selection to predict response to neoadjuvant chemotherapy (Perez-Lopez et al., 2024). However, early fusion ignores distributional differences among modalities and is susceptible to the curse of dimensionality, in which high-dimensional omics data can overwhelm lower-dimensional clinical variables (Wei et al., 2023). The second strategy is late fusion, or decision-level fusion, in which separate models are trained for each modality and their predictions are aggregated by voting or weighted averaging. This design is robust to missing modalities and allows each data type to be analyzed with an appropriate algorithm, such as CNNs for images and multilayer perceptrons for gene-expression data (Bendani et al., 2025). Its main limitation is the reduced ability to capture intermodal interactions, such as associations between specific mutations and imaging phenotypes (Wei et al., 2023; Chang et al., 2025). The third strategy is intermediate, or feature-level, fusion. In this approach, modality-specific subnetworks first extract low-dimensional embeddings, which are then integrated within intermediate network layers (Waqas et al., 2024a). Multimodal autoencoders can map different omics layers into a shared latent space for clustering or classification (Fountzilas et al., 2025; Minoura et al., 2021), whereas moBRCA-net incorporates biological interaction information during feature extraction (Pillai and Meera, 2025). Tensor fusion explicitly models pairwise interactions by computing the outer product of feature vectors from different modalities (Bellini et al., 2023) and may be useful for identifying gene–image associations (Hsu et al., 2026).

As bioinformatics increasingly adopts network-based approaches, graph neural networks (GNNs) and Transformers have become important algorithms for multi-omics fusion. GNNs are particularly well suited to non-Euclidean data such as patient similarity networks (PSNs). For example, MOGONET and the Multi-omics Stacked Fusion Network (MSFN) construct graphs in which nodes represent patients and edges represent omics-based similarity (Wang et al., 2021; Zhang G. et al., 2024). Through graph convolutions, GNNs propagate label information among similar patients and leverage the principle of homophily to improve classification accuracy. In breast cancer survival prediction, MSFN combines residual GCNs with CNNs and outperforms single-omics models (Zhang G. et al., 2024). GNNs can also incorporate biological prior knowledge. The GAIN-BRCA model uses miRNA–mRNA interactions and CpG–mRNA regulatory relationships to construct a graph and learn node weights. This model achieved an AUROC of 0.98 for breast cancer subtype classification, outperformed traditional methods, and identified KRAS as a key prognostic gene in the Luminal A subtype (Patel et al., 2025; Lo et al., 2024).

The Transformer architecture is centered on self-attention, which dynamically weights the features most relevant to a given prediction task (Jiang et al., 2024). In drug-response prediction, such models can learn to prioritize gene-expression modules associated with specific imaging features (Li et al., 2023). For kinase-driven cancers such as HER2-positive breast cancer, attention mechanisms may assign greater weight to omics layers related to kinase signaling pathways (Liu Z. et al., 2026; Wu et al., 2025). Transformers can also treat genomic sequences as text-like inputs, capturing long-range genomic dependencies and improving interpretation of the functional consequences of non-coding mutations (Zhang J. et al., 2025).

## 4 Applications of AI combined with omics in the precision diagnosis of breast cancer

Table 2 summarizes the relative strengths, translational maturity, and representative reported performance of major data modalities used in AI-enabled breast cancer research.

**TABLE 2:** Relative strengths, translational maturity, and representative reported performance of major data modalities for AI-enabled breast-cancer multi-omics integration.

Data modality	Acquisition cost and scalability	Clinical maturity	Interpretability	Representative reported performance	Regulatory readiness	References
Radiomics/conventional imaging	Moderate cost; highly scalable because routine imaging is widely available	Relatively mature, especially for screening and lesion detection	Moderate; affected by scanner type, imaging protocol, segmentation, and feature definition	The Thermalytix Risk Score achieved an AUC of 0.89, outperforming age-based risk assessment with an AUC of 0.68	Relatively advanced among the listed modalities, but external and site-specific validation remains necessary	(Kakileti et al., 2020a)
Digital pathology/pathomics	Moderate cost after slide digitization; scalable in centralized pathology workflows	Increasingly mature for triage, biomarker-adjacent assessment, and prognostic evaluation	Moderate to high when heatmaps or morphology-linked explanations are available	Explainable histology-based models reported balanced accuracy of up to 78% for molecular feature prediction	Emerging; scanner, staining, and laboratory workflow standardization are still required	(Binder et al., 2021)
Genomics/transcriptomics	Moderate to high cost; scalable in academic centers but less routinely available worldwide	Established for selected clinical assays, but less mature for broad AI-based fusion	Moderate; gene sets and pathway annotations support biological interpretation	A multi-omic predictor incorporating molecular and pathological features predicted pCR with an external-validation AUC of 0.87	Moderate for validated clinical assays; lower for exploratory AI-derived signatures	(Sammut et al., 2022)
Proteomics	High cost; limited scalability and substantial sensitivity to platform effects	Mostly research-stage in breast-cancer AI applications	High biological relevance because proteins more directly reflect functional cellular states	Tear proteomics combined with artificial neural networks reported 71.19% sensitivity and 70.69% specificity	Low to moderate; assay standardization and prospective validation are needed	(Lebrecht et al., 2009)
Metabolomics	High cost; limited scalability and strong preanalytical sensitivity	Mostly exploratory or mechanistic in current breast-cancer AI studies	High for pathway-level interpretation and treatment-resistance biology	No consistently validated clinical AUC was available from the cited breast-cancer applications; current evidence mainly supports biomarker discovery and resistance-mechanism analysis	Low; standardized assays and clinical-utility evidence are required	(Rushing et al., 2023)
Liquid biopsy	Moderate to high cost; repeatable and suitable for longitudinal monitoring	Maturing for ctDNA monitoring, but less established for AI-based multi-omics fusion	Moderate; signals reflect tumor burden and resistance evolution but lack spatial context	ctDNA-MRD surveillance showed sensitivity of 0.79 and specificity of 0.98 for postoperative recurrence prediction in a meta-analysis	Moderate for specific ctDNA assays; limited for complex AI-based fusion models	(You et al., 2026)
Single-cell transcriptomics	Very high cost; low throughput and complex analysis	Predominantly exploratory	High cellular resolution, but difficult to summarize in clinically actionable reports	PERCEPTION reported an AUC of 0.776 for treatment-response stratification in the small FELINE breast-cancer cohort	Low; larger validation cohorts and faster workflows are needed	(Sinha et al., 2024)
Spatial transcriptomics	Very high cost; low throughput and complex spatial analysis	Primarily discovery-oriented rather than clinically implemented	High spatial biological interpretability	The cited breast-cancer studies mainly reported spatial niches and drug-sensitivity patterns rather than validated clinical AUC values	Low; large-scale standardization and prospective validation are required	(Yang et al., 2025)

### 4.1 Radiomics

Radiomics-based models have shown promise, mainly in retrospective and multicohort studies, for non-invasively predicting response to immune checkpoint blockade and supporting exploratory biomarker-based patient stratification (Zhao et al., 2023). In mammography, deep-learning models have achieved performance comparable to radiologists for detecting breast masses and calcifications, and multimodal models that incorporate ultrasound images can further improve diagnostic sensitivity (Yan et al., 2026). In MRI research, models integrating multiparametric MRI data have shown high sensitivity for both lesion segmentation and detection (Abdullah et al., 2025). Deep-learning systems applied to mammographic X-ray images can reduce false-positive findings while maintaining radiologist-level accuracy (Wang, 2024). In one study, the system reportedly reduced false-positive and false-negative rates by 17.8% and 5.7%, respectively. Kakileti et al. developed an AI-based thermal-imaging risk prediction framework in which the Thermalytix Risk Score (TRS) achieved an AUC of 0.89, outperforming age-based risk assessment (AUC 0.68) (Kakileti et al., 2020b). This noninvasive and radiation-free approach may be particularly valuable for women with dense breast tissue. By automatically extracting image features, radiomics can identify subtle abnormalities that are difficult to detect visually (He J. et al., 2025). For example, AI can analyze mammograms to identify tumors and calcifications, supporting more efficient diagnosis (Kakileti et al., 2020b). In MRI analysis, radiomics can quantify tumor shape, texture, margins, and heterogeneity.

MRI-based radiomics has shown particular promise for predicting responses to immune checkpoint inhibitors. FDG-PET/CT-based radiomics models can predict TNBC subtypes with high sensitivity and specificity (Krajnc et al., 2021).

Multimodal imaging integration uses AI algorithms to combine complementary imaging modalities and thereby improve predictive accuracy (Ben et al., 2025). Multimodal AI systems can integrate mammography, ultrasound, and MRI data for breast cancer diagnosis (Bendani et al., 2025). One such system achieved an overall AUC of 0.94 and outperformed single-modality approaches. In addition, a cost-effectiveness analysis by a Singapore-based early health technology assessment suggested that AI-based risk prediction followed by selective screening of high-risk women may be cost-effective (Goh et al., 2026).

### 4.2 Genomics

AI algorithms have improved the performance of polygenic risk scores (PRSs) for breast cancer risk assessment (Schuran et al., 2025). An MRI-integrated AI prediction system developed by Li et al. can noninvasively predict the final postoperative residual cancer burden (RCB) grade after two cycles of neoadjuvant chemotherapy and maintains stable diagnostic performance across the three major molecular subtypes of breast cancer. These findings suggest that invasive genomic biomarkers and noninvasive imaging-based models can complement each other in personalized decision-making for neoadjuvant breast cancer therapy (Li et al., 2025). When combined with clinical risk models, PRSs improved net reclassification by 5%–17% across ancestry groups. Machine-learning models also show promise for predicting BRCA1/2 mutations without direct genetic testing. Park H et al. developed a multimodal AI model integrating clinical, imaging, and genomic data to predict BRCA mutations with an AUC of 0.85 (Park et al., 2025). The model identified imaging features associated with BRCA mutations, including increased breast density and structural distortion.

AI algorithms have contributed to the identification of immunogenomic features associated with responses to immune checkpoint inhibitors (Litchfield et al., 2021). Current multi-omics machine-learning models can integrate genomic, transcriptomic, and pathological features to predict pathological complete response (pCR) to neoadjuvant therapy (Wang et al., 2026). In an external validation cohort, one model achieved an AUC of 0.87, outperforming established biomarkers.

### 4.3 Proteomics

Proteomics provides a direct readout of cellular and organismal functional states. In early breast cancer screening, AI-assisted deep proteomics is helping to address the technical challenge posed by the broad dynamic range of blood biomarkers (Joshi et al., 2024). AI-driven proteomics has accelerated the discovery of novel blood-based biomarkers for breast cancer (Mann et al., 2021). Lebrecht et al. further demonstrated the diagnostic potential of tear proteomics. Using surface-enhanced laser desorption/ionization time-of-flight mass spectrometry and artificial neural networks, they identified a 20-biomarker panel with 71.19% sensitivity and 70.69% specificity, suggesting a potential noninvasive screening approach (Lebrecht et al., 2009).

### 4.4 Spatial transcriptomics

Recent advances in spatial transcriptomics provide a systematic framework for defining the physical localization of TME components and generating spatially resolved information relevant to treatment (Elhanani et al., 2023). At single-cell, whole-transcriptome resolution, Spotiphy has revealed spatial and domain-specific differences in tumor–immune interactions within the breast cancer TME (Yang et al., 2025). The intensity of these interactions and the activation of immune-related signaling pathways vary across tumor regions, with pathways related to cell migration and immune evasion specifically upregulated in some spatial domains (Wu et al., 2021). These findings provide spatial evidence for mechanisms of immune evasion in breast cancer (Yang et al., 2025). They also challenge the conventional view that the tumor core is uniformly the most immunosuppressive region, suggesting that the core may have a strong capacity to recruit CD8^+^ T cells (Jackson et al., 2020). These CD8^+^ T cells can penetrate the collagen matrix through COL6A3–ITGB1 ligand–receptor interactions and form a honeycomb-like immune defense structure within the tumor core. Such spatial patterns may help explain heterogeneous immunotherapy responses among patients with the same pathological subtype and may support the identification of responsive subgroups and therapeutic targets. In addition, Spotiphy identified an FSTL1+ fibroblast subpopulation whose spatial clustering pattern correlated with the efficacy of PD-1 inhibitors, suggesting a potential spatial biomarker for immunotherapy response (Friedman et al., 2020).

Starfysh integrates spatial transcriptomics with histological data to reveal heterogeneous tumor–immune hubs. Using deep generative modeling and archetype analysis, it characterizes tissue-specific cellular states without requiring single-cell reference data. In breast cancer, Starfysh identified spatial hubs composed of patient- and disease-specific cell populations, showing how metabolic reprogramming can shape immunosuppressive niches in aggressive disease (He S. et al., 2025). Spatial single-cell transcriptomic analysis has also identified potential biomarkers for PD-1 blockade. Using Xenium technology to analyze the TME across breast cancer subtypes, investigators found CD274/CD80 interactions between macrophages and T cells and observed that effector T cells, proliferating T cells, and macrophages were located closer to tumor cells in responders. These features may serve as biomarkers for immunotherapy response.

### 4.5 Liquid biopsy

Traditional tissue biopsies are invasive and provide only a static snapshot from a localized tumor region (Lone et al., 2022); they cannot fully capture spatial heterogeneity or dynamically monitor resistance mechanisms driven by clonal selection during treatment (Yang et al., 2023). AI-integrated liquid biopsy is reshaping approaches to early screening and recurrence monitoring in breast cancer (Luo et al., 2025). Circulating tumor DNA (ctDNA) provides a minimally invasive window into the tumor genomic landscape (You et al., 2026). AI-assisted computational models may enhance ctDNA-based treatment monitoring by integrating longitudinal molecular signals with clinical response data. In metastatic breast cancer, shallow whole-genome sequencing of ctDNA combined with Bayesian machine-learning modeling has been shown to predict treatment response and progression-free survival, outperforming CA15-3-based assessment. In the neoadjuvant setting, serial ctDNA dynamics and AI-based quantification of tumor-infiltrating lymphocytes may provide complementary information for predicting pathological response and post-treatment risk stratification (Beddowes et al., 2025). Machine-learning methods have also identified miRNA signatures with high diagnostic accuracy; for example, an 8-miRNA panel achieved an AUC of 0.915, 82.3% accuracy, 72.2% sensitivity, and 91.5% specificity for breast cancer detection (Tao et al., 2025). AI algorithms can further distinguish cancer types and predict drug efficacy, supporting personalized medicine (Bera et al., 2019). Table 3 summarizes representative omics and AI-enabled multimodal studies in breast cancer, including their clinical relevance, evidence status, and translational limitations.

**TABLE 3:** Representative AI-enabled omics and multimodal studies in breast cancer: clinical utility, evidence status, and translational limitations.

Omics/data modality	Main clinical task	AI/computational approach	Key finding	Evidence status and main limitation	Clinical-readiness interpretation	References
Thermal radiomics/infrared imaging	Radiation-free pre-screening and risk stratification	Thermal radiomics with machine-learning risk scoring	The Thermalytix Risk Score achieved an AUC of approximately 0.89–0.90 for breast cancer risk prediction	Observational diagnostic study; not a replacement for mammography, ultrasound, MRI, or histopathology	Promising as an adjunctive pre-screening or risk-stratification tool, especially in radiation-free or low-resource settings	(Kakileti et al., 2020a)
FDG-PET/CT radiomics	Tumor characterization and molecular-status prediction	Ensemble-learning radiomics/holomic models	FDG-PET/CT radiomics supported breast tumor characterization, receptor-status prediction, proliferation assessment, and triple-negative tumor identification	Retrospective imaging-based study; limited sample size and variable performance across targets	More suitable for biological characterization and response-assessment support than routine population-level screening	(Krajnc et al., 2021)
Integrated multi-omics	Prediction of neoadjuvant therapy response/pCR	Machine-learning integration of clinical, genomic, transcriptomic, pathological, and microenvironmental features	The multi-omic model predicted pCR in an external validation cohort with an AUC of 0.87	High-quality translational study with external validation; prospective clinical implementation remains lacking	One of the strongest clinical examples of AI-enabled multi-omics, but not yet a routinely deployed tool	(Sammut et al., 2022)
Tissue proteomics/mass spectrometry imaging	ER, PR, HER2, and TNBC-related molecular characterization	Mass spectrometry imaging with machine-learning classification	The study analyzed 1,191 breast cancer samples and evaluated receptor-status classification	Large retrospective tissue-based cohort; requires specialized MSI infrastructure	Useful as molecular characterization support, but not a routine replacement for IHC/FISH.	(Gonçalves et al., 2023)
Pathomics/explainable ML	Prediction of molecular, morphological, and immune-related features from histology	Explainable machine learning linking histology with molecular and clinical features	Molecular predictions reached balanced accuracies up to 78%, with higher accuracy in selected subgroups	Retrospective explainable-AI study; subgroup performance may not generalize	Useful for interpretability and molecular inference from routine pathology, but not a substitute for molecular assays	(Binder et al., 2021)
Pathomics/H&E whole-slide images	Prediction of biomarkers, immune signatures, and prognosis-associated subtypes	BBMIL/multi-instance deep learning	BBMIL predicted classical biomarkers, immunotherapy-associated gene signatures, and prognosis-associated subtypes from H&E slides	Multicentre retrospective study; performance varies across biomarkers	Strong example of pathology-based triage and biomarker prioritization, but not definitive molecular diagnosis	(Zhang et al., 2025b)
Single-cell transcriptomics	Prediction of drug response and resistance	Transfer-learning framework using bulk and single-cell transcriptomic profiles	PERCEPTION predicted treatment response using single-cell transcriptomic information in preclinical and clinical cohorts	Proof-of-concept precision-oncology pipeline; prospective treatment-assignment validation is lacking	Important for future precision oncology, but currently investigational	(Sinha et al., 2024)
Single-cell ligand–receptor profiling	TNBC immunotherapy-response stratification and prognosis	Ligand–receptor interaction scoring/LR.score	A ligand–receptor-based score was proposed to identify an immunotherapy-responsive TNBC subtype and prognostic signature	Computational and retrospective validation evidence; prospective ICI-treated TNBC validation is needed	Useful as hypothesis-generating biomarker evidence for the immunotherapy section	(Chen et al., 2025b)
Spatial transcriptomics	Spatial mapping of tumor–TME interactions	Generative spatial transcriptomic framework	Spotiphy enables spatial visualization of cellular localization and transcriptomic profiles across tissue sections	High-resolution computational study; mainly mechanistic and discovery-oriented	Best used to interpret spatial heterogeneity and tumor–TME organization, not direct clinical decision-making	(Yang et al., 2025)
Spatial transcriptomics + histology	Identification of spatial tumor–immune hubs	Deep generative model integrating spatial transcriptomics and histology	Starfysh identified heterogeneous spatial hubs and tumor–immune niches across tissue contexts	Strong methodological study; clinical endpoints are indirect	Suitable for mechanistic and translational-frontier discussion, not routine clinical implementation	(He et al., 2025b)
Metabolomics/drug resistance	Mechanistic analysis of chemotherapy resistance in TNBC	Metabolomic profiling with pathway-level interpretation	The study identified metabolic changes after acquisition of doxorubicin resistance in TNBC cell models	Cell-line mechanistic study; not patient-level predictive evidence	Keep as a mechanistic resistance example, not as clinical decision-support evidence	(Rushing et al., 2023)

## 5 Applications of AI in predicting immunotherapy response and drug sensitivity

### 5.1 Pathomics

Breast cancer prognosis assessment and drug-sensitivity prediction no longer rely solely on TNM staging. By analyzing pathological images, single-cell microenvironmental differences, spatial cell-distribution patterns, and metabolic changes, AI can support multidimensional precision prediction systems (Boehm et al., 2022). In BioFusionNet, the integration of histopathological, genetic, and clinical data increased the C-statistic for survival prediction to 0.77, demonstrating the value of combining AI-derived histopathological features with clinical variables (Mondol et al., 2024). AI models can also predict molecular subtypes directly from hematoxylin and eosin (H&E)-stained images. BBMIL, a deep-learning algorithm developed for this purpose, predicts classical biomarkers, immunotherapy-related genomic features, and prognosis-related subtypes with high accuracy and outperforms existing methods in independent cohorts (Zhang X. et al., 2025).

### 5.2 Single-cell sequencing

Traditional bulk sequencing can obscure cellular heterogeneity within tumors. By combining AI with scRNA-seq, researchers can characterize differences among TME cell subpopulations and improve drug-sensitivity prediction (Zhang et al., 2021). PERCEPTION (Personalized Single-Cell Expression-Based Planning for Treatments in Oncology) is a transfer-learning AI model. It is first pretrained on large cell-line drug-response datasets, such as GDSC, to learn general relationships between transcriptomic features and drug sensitivity (Sinha et al., 2024), and is then fine-tuned with patient scRNA-seq data to address the limited availability of single-cell clinical datasets. A major strength of PERCEPTION is its ability to detect residual drug-resistant subclones. Even when a tumor appears broadly sensitive to a drug, the presence of a small resistant subclone may predict poor outcome (Maeser et al., 2024; Mao et al., 2025). In a breast cancer cohort, the model tracked resistance evolution during treatment and predicted treatment-failure risk for agents such as CDK4/6 inhibitors (Sinha et al., 2024). AI analysis has also shown that intercellular ligand–receptor communication networks are important determinants of prognosis and drug sensitivity (Lee et al., 2024). For example, AI-driven clustering in TNBC identified immune-active (Clust1) and immune-exhausted (Clust2) microenvironmental subtypes (Chen Y. et al., 2025).

### 5.3 Spatial transcriptomics

Spatial transcriptomics adds spatial coordinates to gene-expression data. AI applications in this area mainly aim to define spatial cell-distribution patterns and clarify their influence on treatment response (Hu et al., 2021). Computational analysis of spatial transcriptomic data has been used to identify therapeutic clusters in breast cancer, defined by spatially resolved predicted drug-sensitivity profiles (Jiménez-Santos et al., 2024). Drug-sensitivity scores, such as the Beyondcell Score, correlate with radial distance from the tumor core. Peripheral tumor cells often have better blood supply and greater immune infiltration, making them more sensitive to certain therapies, whereas core-region cells are more frequently hypoxic and drug resistant (Xun et al., 2024). AI models also suggest that tumor clones with identical genotypes may show markedly different drug sensitivities because of their spatial context, such as localization within ducts or stroma (Jiménez-Santos et al., 2024).

### 5.4 Metabolomics

Metabolic reprogramming is a hallmark of cancer. In metabolomics, AI-based approaches assess treatment sensitivity by analyzing the metabolic profiles of tumor tissues (Colak et al., 2025). For example, doxorubicin-resistant TNBC cells exhibit marked metabolic rewiring, with clear differences in metabolic intensity and pathway activity compared with sensitive cells. These differences include accumulation of long-chain acylcarnitines and reduction of short- and medium-chain acylcarnitines (Rushing et al., 2023). This pattern suggests decoupling between glycolysis and fatty acid oxidation (FAO) (Koundouros and Poulogiannis, 2020), in which resistant cells incompletely degrade fatty acids and accumulate intermediate metabolites (Stine et al., 2022). Such metabolic alterations can help AI models distinguish sensitive from resistant strains with high accuracy (Baptista et al., 2021). AI analysis also revealed that metabolite levels in sensitive cells fluctuate with drug concentration, whereas resistant cells exhibit a rigid or locked metabolic state that no longer responds dynamically to drug stimulation (Hanahan, 2022). This metabolic rigidity may indicate poor prognosis (Rushing et al., 2023). Algorithms such as Random Forest and LightGBM (Reel et al., 2021), together with multi-omics feature selection (MOFS), can be used to construct metabolite-based predictive models. AI has identified glutamate, acetylcarnitine, and choline phosphate as key predictors of drug response (Schmidt et al., 2021). For example, choline phosphate is higher in sensitive cells but decreases as resistance develops (Martínez-Reyes and Chandel, 2021). These findings suggest that monitoring metabolic activity in blood or tissue may enable dynamic assessment of treatment sensitivity (Schmidt et al., 2021). The integration of SHAP explainability with metabolomics links computational predictions to biological interpretation and improves clinical utility (Lundberg et al., 2020). XAI-driven metabolomic machine learning may therefore improve breast cancer diagnosis while revealing metabolic dysregulation associated with disease progression (Guldogan et al., 2025).

## 6 Technical bottlenecks and information silos in omics-based assessment of breast cancer immunotherapy

Clinical maturity should be interpreted unevenly across AI technologies. AI assistance for mammographic screening and selected digital-pathology tasks is closest to routine implementation because these workflows use standardized clinical inputs, established diagnostic endpoints, and increasingly defined quality-management processes (Gommers et al., 2026). In contrast, spatial transcriptomics-guided prediction, single-cell AI drug-response modeling, and multi-omics immunotherapy-response systems remain emerging research technologies (Add et al., 2024; Gulati et al., 2025). These approaches provide mechanistic insight and may identify candidate biomarkers, but they generally require fresh or specialized tissue processing, complex computational pipelines, longer turnaround times, and prospective evidence that treatment decisions based on model outputs improve outcomes (Prelaj et al., 2024).

Although single-omics technologies have substantially advanced the molecular characterization of breast cancer and the development of targeted therapies, single-dimensional approaches face important bottlenecks when applied to the complex TME and heterogeneous immune responses to immunotherapy (Mora et al., 2021; Subramanian et al., 2020). Single-omics data therefore have intrinsic limitations. Owing to their different detection principles, each omics layer captures only part of the biological system (Baysoy et al., 2023; Picard et al., 2021):

Genomics: whole-exome sequencing (WES) can identify TMB and specific mutations such as BRCA1/2 and PIK3CA, but static DNA-level alterations cannot be directly equated with protein expression or functional pathway activation (Krug et al., 2020; Mani et al., 2022). Genomic data alone also cannot adequately reflect post-transcriptional modification and epigenetic regulation, resulting in a genotype–phenotype gap (Vasan et al., 2019).

Transcriptomics and Spatial Transcriptomics: There is often a time lag and a nonlinear relationship between RNA levels and the abundance of proteins that ultimately perform biological functions (Buccitelli and Selbach, 2020). Furthermore, while spatial transcriptomics preserves information on the spatial location of cells, it currently faces challenges such as high costs, the need for improved single-cell resolution, and a lack of longitudinal dynamic monitoring capability, making it difficult to implement on a large scale in clinical cohorts (Marx, 2021).

Proteomics: As the omics discipline closest to the agents of biological activity, proteomics has a natural advantage in identifying immune targets. However, due to technical limitations (such as the lack of signal amplification techniques similar to PCR) (Timp and Timp, 2020), it lacks sufficient sensitivity for detecting extremely low-abundance cytokines or immunoregulatory proteins in the TME, and the data are often noisy (Suhre et al., 2021).

Radiomics: Radiomics enables non-invasive, macroscopic assessment of the heterogeneity of the entire tumor and its surrounding stroma (van Timmeren et al., 2020); however, it essentially involves the extraction of macroscopic physical features and lacks microscopic resolution at the molecular and cellular levels (Lipkova et al., 2022). Furthermore, imaging features are highly susceptible to interference from different imaging devices, scanning parameters, and reconstruction algorithms, resulting in poor cross-center generalization capabilities of AI models (Bera et al., 2019).

Liquid biopsy: ctDNA or circulating tumor cells (CTCs) offer non-invasive methods for dynamic monitoring (Xu et al., 2025); however, their abundance is extremely low in patients with early-stage breast cancer, posing a risk of false negatives (Xi et al., 2024). More importantly, molecular signals in the blood lack spatial context, making it difficult to trace the exact spatial origin of mutated clones and their interactions with local immune cells (Ignatiadis et al., 2021).

Furthermore, compared to multi-omics, single-omics approaches suffer from more systemic limitations, such as the lack of a comprehensive perspective (Hasin et al., 2017)—analogous to a limited, unidimensional view of a complex biological system (Olivier et al., 2019). Tumor immunology is a complex system involving multi-level, multi-dimensional interactions. Compared to multi-omics, the central limitation of single-omics lies in its “information silo” effect (Boehm et al., 2022). A single omics approach cannot capture the complex biological networks involved (Havel et al., 2019); that is, the efficacy of immunotherapies (such as PD-1/PD-L1 inhibitors) depends not only on target expression but also on regulation by multiple factors, including antigen presentation, metabolic reprogramming, and angiogenesis (Hegde and Chen, 2020). A single omics approach cannot reconstruct the complete biological network spanning from “upstream gene regulation” to “downstream metabolic/phenotypic changes” (Pinu et al., 2019). When building AI predictive models, single-omics data often encounter performance bottlenecks due to the limited dimensionality of its features (Zitnik et al., 2019). For example, relying solely on radiomics or genomics to predict pCR to immunotherapy in breast cancer may lead to misclassification in complex cases (such as immunological pseudo progression) (Ran D. et al., 2025). AI analysis based on a single data modality may capture “associated” features related to prognosis rather than true “driving” biological mechanisms (Moor et al., 2023; Chen et al., 2022). Cross-validation across multi-omics data—such as the alignment of imaging features with spatial transcriptomics—is the key approach to eliminating false-positive associations and identifying robust biomarkers (Lipkova et al., 2022).

## 7 AI-driven multimodal data fusion and clinical applications

Figure 2 illustrates complementary combinations of omics and imaging modalities and their potential applications in AI-enabled breast cancer research. In the face of the information silos created by omics data, AI—particularly deep learning and GNNs—has demonstrated strong capabilities in high-dimensional data mapping and feature fusion (Wang et al., 2021). By constructing multimodal fusion networks, AI can break down the scale barriers between the microscopic molecular, mesoscopic cellular, and macroscopic tissue levels, providing a feasible solution for assessing immunotherapy sensitivity and stratifying prognosis, a finding supported by research evidence from other types of cancer (Sammut et al., 2022; Vanguri et al., 2022).

**FIGURE 2:**
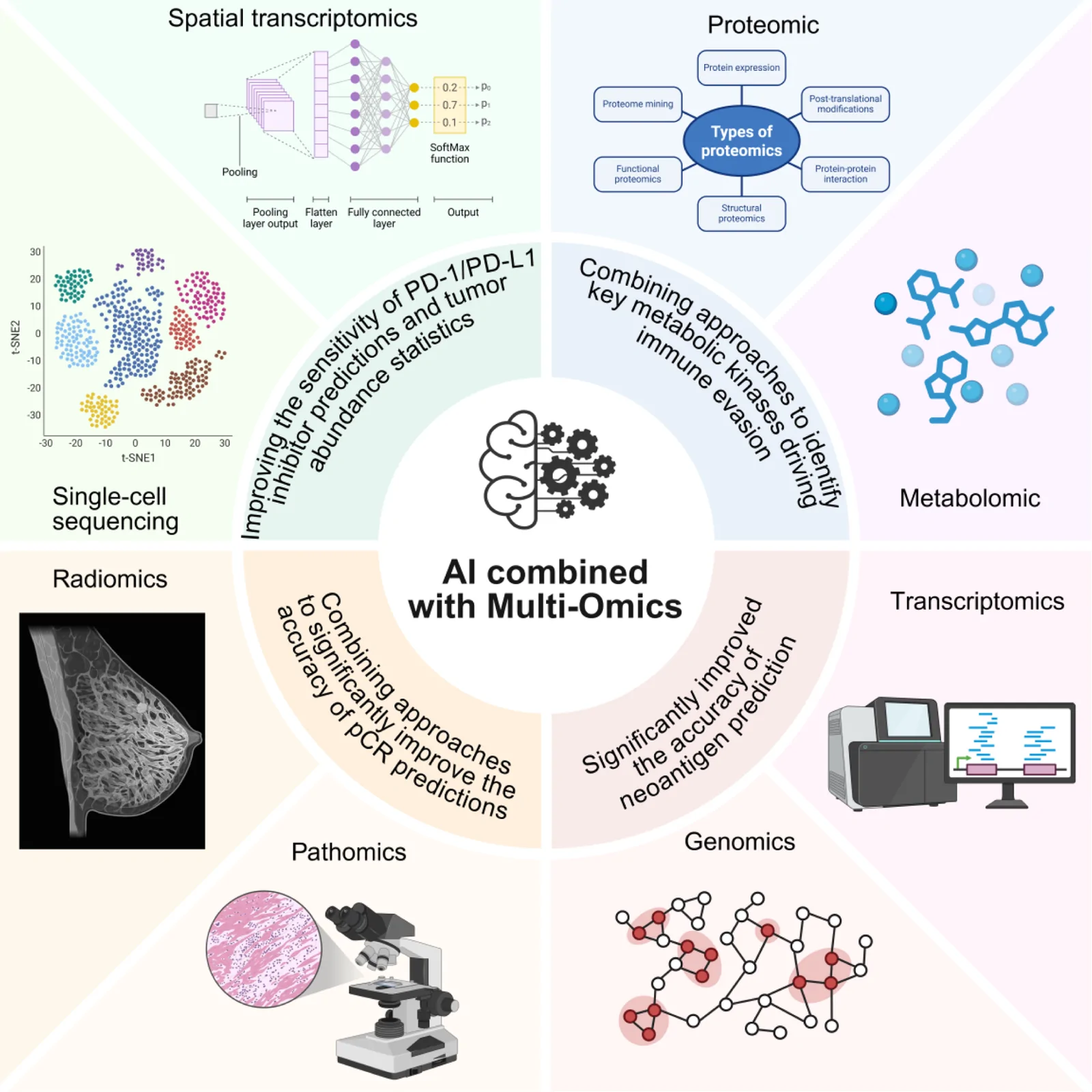
AI combined with multi-omics. This figure illustrates how AI combined with spatial transcriptomics and single-cell sequencing, proteomics and metabolomics, radiomics and pathomics, and genomics and transcriptomics, enhances clinical diagnostic capabilities.

### 7.1 Spatial transcriptomics combined with single-cell sequencing provides high-resolution insights into the tumor immune microenvironment

The spatial heterogeneity of immune cells in the TME is a key factor determining the response to immunotherapy in breast cancer, particularly in TNBC (Xin et al., 2025). scRNA-seq offers single-cell resolution but lacks spatial coordinates, whereas spatial transcriptomics preserves tissue structure but is limited by cellular mixing at sequencing spots (Shi et al., 2025). AI algorithms based on deconvolution models using graph convolutional networks or autoencoders can bridge the strengths of both approaches, specifically by mapping the characteristics of cell subpopulations defined by scRNA-seq as prior knowledge onto spatial transcriptomics data, AI can accurately infer the absolute abundance and spatial proximity interaction networks of different immune cells (such as exhausted CD8^+^ T cells and M2 macrophages) within each spatial location (Liao et al., 2021). This multi-omics integration not only enables the precise identification of tertiary lymphoid structures (TLS) closely associated with immunotherapy response, but also predicts patient sensitivity to PD-1/PD-L1 inhibitors through spatial communication networks, thereby elevating the distinction between “cold tumors” and “hot tumors” from a simple statistical measure of abundance to the level of spatial topology (Keren et al., 2018).

### 7.2 Proteomic-metabolomic integration reveals functional phenotypes and vulnerabilities in metabolic reprogramming

Genes and transcripts serve as biological instructions, while proteins and metabolites act as “molecular snapshots” that ultimately carry out biological processes and reflect transient phenotypes (Pavlova et al., 2022). Breast cancer often undergoes significant metabolic reprogramming under immunotherapeutic stress—such as enhanced glycolysis or lipid metabolism abnormalities—which suppresses the function of infiltrating immune cells (Arner and Rathmell, 2023). AI, leveraging machine learning frameworks such as multi-module DNNs (e.g., multi-channel variational autoencoders) or orthogonal partial least squares (OPLS), can effectively filter out noise in high-throughput proteomic and metabolomic data to extract “protein-metabolite” co-expression modules that change in concert. This integration not only aids in identifying key metabolic kinases driving immune evasion but also uncovers composite biomarker panels with high clinical accessibility in blood or tissues, providing new metabolic intervention targets for overcoming resistance to immunotherapy (Picard et al., 2021).

### 7.3 Cross-scale prognostic prediction and improved diagnostic performance through the integration of radiomics and pathology

At the macroscopic and microscopic levels of clinical morphology, imaging modalities such as MRI and ultrasound capture the tumor’s overall blood supply, invasion boundaries, and macroscopic heterogeneity (Rajpurkar et al., 2022), while digital pathology techniques such as panoramic slide images and WSI provide subcellular-level details on cell morphology, mitotic figures, and stroma-to-tumor ratios (Diao et al., 2021). AI architectures based on Multiple Instance Learning (MIL) and Visual Transformers (ViT) enable deep cascaded fusion of “macroscopic imaging–microscopic pathology” features (Chen et al., 2022). Studies have shown that this cross-scale multimodal AI model significantly outperforms any single-modality approach in predicting pCR and disease-free survival (DFS) following neoadjuvant therapy for breast cancer (Bera et al., 2022). Furthermore, by generating high-precision multimodal risk scores, clinicians can directly plot smoother and more distinct survival curves (Kaplan-Meier survival curves) in analytical environments, enabling non-invasive, efficient preoperative individualized survival stratification and optimizing the clinical decision-making process (Kline et al., 2022).

### 7.4 Genomics and transcriptomics: decoding the immunogenicity of neoantigens and dynamic immune evasion

Genomic sequencing approaches, such as whole-exome sequencing, can reveal the mutational substrate that may give rise to tumor neoantigens (Lang et al., 2022); however, this potential must be translated into actual expression at the RNA level and effectively processed and presented on HLA molecules at the cell surface to trigger substantial T-cell killing (Xie et al., 2023).AI-driven deep learning algorithms combining machine learning with attention-based DNNs perform high-dimensional joint mapping of the static DNA mutation landscape of the genome with the dynamic expression matrix of the transcriptome (Chowell et al., 2022). This computational framework integrates gene expression levels, mutation allele frequencies, Human Leukocyte Antigen (HLA) affinity, and TCR repertoire characteristics (Pham et al., 2024). By leveraging this cross-omics data dimensionality reduction and feature alignment, the AI can filter out large amounts of “invalid mutation” noise—such as unexpressed or non-presentable mutations—significantly improving the accuracy of neoantigen prediction (reducing false positives), but also systematically elucidated how tumor cells achieve immune evasion through post-transcriptional modifications or loss of heterozygosity (LOH) in HLA (Wells et al., 2020). The integration of this dimension represents an important computational foundation for developing personalized mRNA cancer vaccines for breast cancer and identifying potential beneficiaries of immune checkpoint inhibitors (Barbier et al., 2022; Baxi et al., 2022). Table 4 compares AI integration strategies for breast cancer multi-omics and multimodal analysis, highlighting their applications, advantages, limitations, and clinical readiness.

**TABLE 4:** Comparative assessment of AI integration architectures for multi-omics and multimodal analysis in breast cancer.

AI integration architecture/strategy	Typical input modalities	Main use in breast cancer research	Key advantage	Main limitation	Clinical-readiness interpretation	References
Preprocessing and harmonization before fusion	Genomics, transcriptomics, proteomics, metabolomics, imaging, pathology, and clinical variables	Reducing technical noise, correcting batch effects, aligning heterogeneous datasets, and imputing missing modalities before model training	Improves data comparability across cohorts and platforms; reduces non-biological variation before downstream prediction	Performance depends strongly on preprocessing choices; overcorrection may remove true biological signals; missing-value imputation may introduce artificial patterns	Essential methodological step, but not a clinical model by itself; should be reported transparently in AI-omics studies	(Hsu et al., 2026; Perez-Lopez et al., 2024; Fountzilas et al., 2025)
Early fusion/concatenation	Feature-selected genomic, transcriptomic, pathological, imaging, and clinical variables	pCR prediction, molecular subtype classification, prognosis modeling, and treatment-response prediction	Simple, transparent, and easy to implement; allows direct integration of multiple feature types into one model	Susceptible to the curse of dimensionality; high-dimensional omics may overwhelm low-dimensional clinical variables; sensitive to missing modalities and distributional differences	Useful for exploratory and moderate-scale studies, but requires strict feature selection, dimensionality reduction, and external validation before clinical translation	(Jiang et al., 2026; Sammut et al., 2022; Waqas et al., 2024b)
Late fusion/decision-level fusion	Separate models for imaging, pathology, omics, and clinical data, followed by voting, averaging, or weighted ensemble prediction	Combining predictions from modality-specific models for diagnosis, prognosis, and treatment-response assessment	More robust to missing modalities; each data type can be modeled with the most appropriate algorithm, such as CNNs for images and MLPs for gene-expression data	Limited ability to capture biological interactions between modalities, such as genotype–imaging or pathology–transcriptome relationships	Practical for real-world heterogeneous data, but less suitable when the scientific aim is to uncover cross-modal mechanisms	(Acosta et al., 2022; Wei et al., 2023; Waqas et al., 2024b)
Intermediate fusion/feature-level fusion	Modality-specific embeddings from omics, imaging, pathology, and clinical data	Integrative subtype discovery, pCR prediction, survival prediction, and biomarker discovery	Captures shared latent representations and cross-modal interactions better than early or late fusion	Less interpretable than simple models; requires larger datasets and careful tuning; risk of overfitting remains high in small breast cancer cohorts	Promising for translational research, but clinical adoption requires interpretable outputs and prospective multicenter validation	(Minoura et al., 2021; Zhang et al., 2024a; Sammut et al., 2022; Waqas et al., 2024b)
Network-aware integration/graph neural networks	Patient similarity networks, gene–gene networks, miRNA–mRNA interactions, CpG–mRNA regulatory relationships, and multi-omics features	Breast cancer subtype classification, survival prediction, and identification of network-level biomarkers	Incorporates biological relationships and non-Euclidean data structures; can model patient similarity and regulatory interactions	Results depend on graph-construction assumptions; biological priors may be incomplete or biased; external reproducibility can be difficult	Valuable for mechanism-oriented biomarker discovery, but not yet ready as a stand-alone clinical decision system	(Wang et al., 2021; Zhang et al., 2024a; Patel et al., 2025)
Transformer/attention-based multimodal learning	Genomic sequences, transcriptomic profiles, imaging features, pathology features, longitudinal clinical data, and treatment-response data	Drug-response prediction, biomarker prioritization, sequence-based interpretation, and multimodal prognosis modeling	Self-attention can identify task-relevant features and model long-range dependencies; useful for complex high-dimensional data	Data-hungry and computationally intensive; interpretability remains limited; may overfit when training cohorts are small	Promising but still research-stage in breast cancer multi-omics; should be presented as an emerging architecture rather than a clinically established method	(Liu et al., 2026b; Wu et al., 2025; Zhang et al., 2025a)
Spatial multimodal integration	Spatial transcriptomics, scRNA-seq, H&E histology, multiplex protein imaging, and spatial coordinates	Mapping tumor–immune niches, identifying spatial TME patterns, and exploring immune-evasion mechanisms	Preserves spatial context that is lost in bulk omics; links cellular states with tissue architecture and local immune interactions	Expensive, technically complex, and usually cross-sectional; many studies remain mechanistic or exploratory; clinical endpoints are often indirect	Best suited for mechanistic discovery and biomarker hypothesis generation, not routine clinical decision-making at present	(Yang et al., 2025; He et al., 2025b; Jiménez-Santos et al., 2024)
Explainable AI-assisted integration	Radiomics, pathomics, metabolomics, clinical variables, and selected molecular biomarkers	Interpreting model decisions, identifying clinically meaningful features, and improving clinician trust	Enhances transparency by linking predictions to interpretable features such as tissue morphology, immune infiltration, or metabolite contribution	Explanations may be model-dependent and not always biologically causal; SHAP or attention weights can be overinterpreted	Important for clinical translation, but should complement rather than replace biological validation	(Binder et al., 2021; Lundberg et al., 2020; Guldogan et al., 2025; Ghasemi et al., 2024)

## 8 AI-driven multi-omics analysis for predicting immunotherapy response and drug-resistance mechanisms

The key challenge in current breast cancer immunotherapy is predicting which patients will benefit from PD-1/PD-L1 inhibitors (Fang et al., 2024; Chang et al., 2024). AI-based composite models may complement conventional biomarkers such as TMB and PD-L1 expression, but their role in breast cancer remains investigational and requires prospective validation (Fm et al., 2023). The LORIS multimodal predictive model is an interpretable AI model based on logistic regression that integrates clinical features (age, stage), TMB, and specific genomic features. Although LORIS was developed and validated in a pan-cancer setting rather than breast cancer-specific cohorts, it illustrates how interpretable multimodal models may complement TMB and PD-L1 for ICB-response prediction. LORIS outperformed TMB alone in predicting response to immune checkpoint blockade (ICB). LORIS outperformed TMB, the PD-L1 tumor proportion score, and several previous machine learning models on nearly all metrics across both internal and external datasets; both TMB and the PD-L1 tumor proportion score are known biomarkers of ICB efficacy (Tavolara et al., 2025). More importantly, as a “white-box” model, LORIS clearly demonstrates the contribution of each feature to the final risk score, significantly enhancing clinicians’ confidence in the model. In the context of neoadjuvant therapy, a multi-omics integrated machine learning model—developed by combining tumor mutation, immune cell infiltration, and pathological image features—successfully predicted the pCR rate of TNBC patients receiving immunotherapy combined with chemotherapy (Cui and Zhang, 2021). The model may help identify patients who are more likely to benefit from treatment and generate hypotheses for future combination strategies (Sammut et al., 2022).

The metabolic environment acts as a “regulator” of immune cell function. By analyzing metabolomics data, an AI model has uncovered the metabolic roots of immune resistance in breast cancer (Yin et al., 2026). By analyzing plasma metabolomic profiles using deep learning, researchers identified a set of metabolic biomarkers comprising polyamines, glycerophosphocholine, and pentosidine. Abnormal levels of these metabolites were highly correlated with residual disease (RCB-II/III) following neoadjuvant chemotherapy. In particular, the accumulation of polyamines is known to inhibit T-cell proliferation and function (Wik et al., 2025; Colak et al., 2025). The “necrotic margin” feature identified by the radiomic model is, in fact, a macroscopic manifestation of tumor hypoxia. Hypoxia leads to the activation of the HIF-1α pathway and the accumulation of adenosine, which directly suppresses the activity of cytotoxic T cells. By integrating imaging-derived hypoxia features with metabolic data, the AI model can accurately identify patient subgroups that require combined anti-hypoxia therapy (such as adenosine receptor antagonists) (Song et al., 2025). Figure 3 provides an overview of the links between data modalities, AI integration strategies, clinical endpoints, and requirements for clinical translation in breast cancer.

**FIGURE 3:**
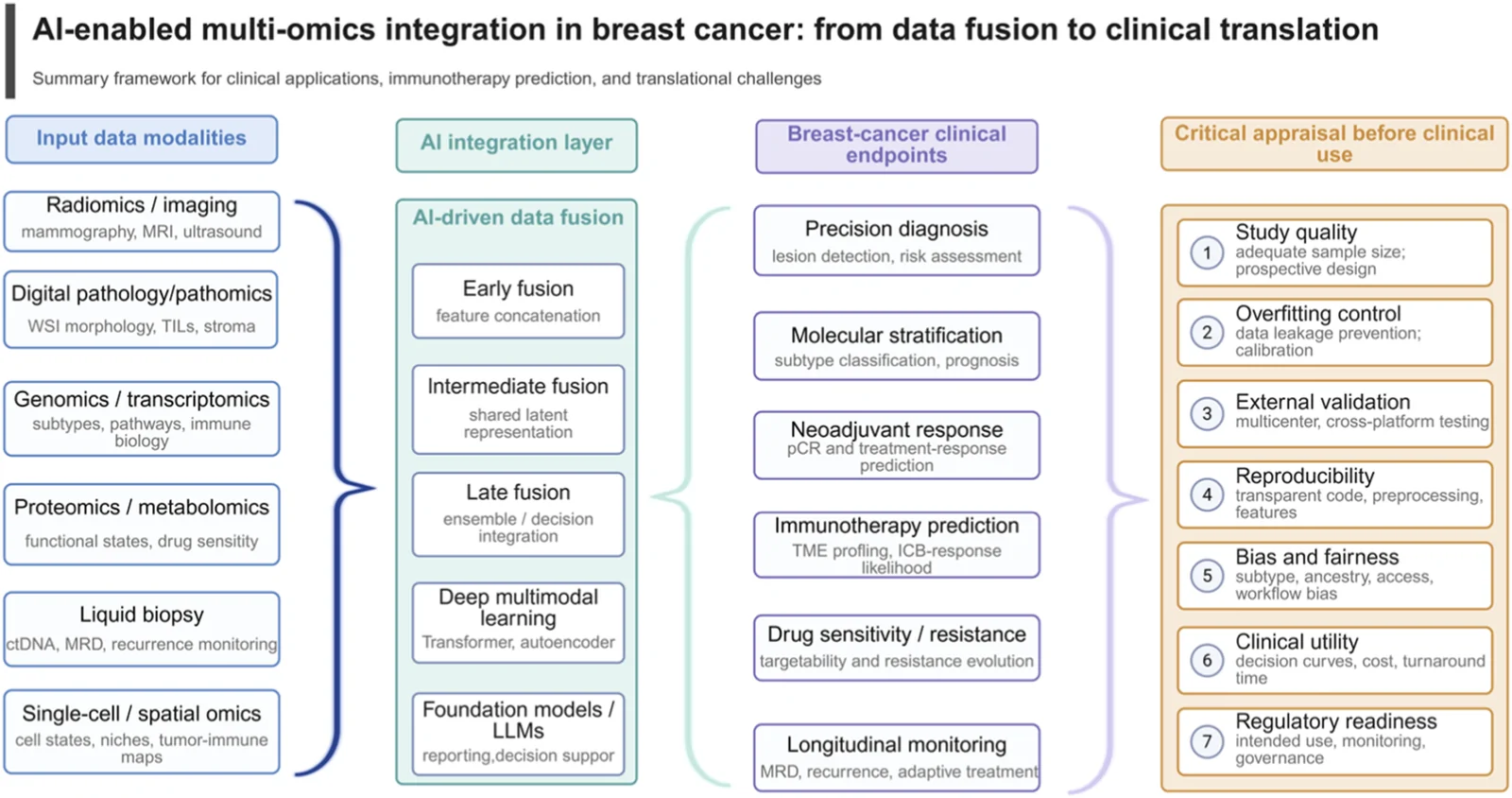
Summary framework of AI-enabled multi-omics integration in breast cancer. This figure summarizes the major data modalities, AI-based fusion strategies, breast-cancer clinical endpoints, and clinical-readiness checkpoints for AI-enabled multi-omics integration. It emphasizes that high model performance alone is insufficient for clinical adoption, and that robust translation requires external validation, reproducibility, interpretability, fairness, workflow feasibility, and regulatory-grade evidence.

## 9 Discussion: critical appraisal and translational challenges

Although the integration of AI and multi-omics has shown substantial potential in breast cancer targeted therapy and immunotherapy research, its translation into routine clinical decision-support tools still faces major challenges across multiple domains (Varoquaux and Cheplygina, 2022). At present, the translation gap is mainly related not only to technical and regulatory barriers, but also to the methodological quality, reproducibility, and generalizability of existing AI-enabled multi-omics studies (Vandereyken et al., 2023). Therefore, beyond summarizing reported model performance, it is necessary to critically evaluate cohort size, validation strategy, overfitting risk, potential bias, clinical utility, and translational readiness (Lekadir et al., 2025).

### 9.1 Critical appraisal of study quality and generalizability

A key limitation of current AI-enabled multi-omics studies in breast cancer is that many models are developed using retrospective datasets, relatively small cohorts, or single-center populations. This issue is particularly important for high-dimensional omics and deep-learning models, in which the number of molecular, imaging, pathological, or spatial features may substantially exceed the number of available patients (Tsegaye et al., 2025). Under these conditions, models may achieve high performance in internal validation while learning cohort-specific noise, platform effects, or preprocessing artifacts rather than stable biological signals (Howard et al., 2021). Therefore, reported performance metrics such as accuracy, AUC, or C-index should be interpreted cautiously unless they are supported by independent external validation, calibration analysis, and clinically meaningful endpoints (Collins et al., 2024).

External validation and reproducibility remain inconsistent across the field.AI models often perform exceptionally well on training datasets but see a sharp drop in performance on external datasets. This is primarily due to the technical batch effect. Domain Adversarial Adaptation designs an adversarial network to force the feature extractor to learn features driven by biological differences across different datasets, thereby ignoring variations caused by technical platforms (such as Illumina and Ion Torrent). This approach has demonstrated significant improvements in robustness in breast cancer subtyping across the TCGA and METABRIC cohorts178. Next-generation AI models (such as Visual Neural Networks) map neural network layers one-to-one with biological pathways (such as the apoptosis pathway and the PI3K pathway). When the model predicts a “high risk,” it can simultaneously identify which signaling pathway is causing the abnormality, thereby directly guiding targeted drug therapy52,179.

Nevertheless, improving generalizability requires more than technical domain adaptation (Kolla and Parikh, 2024). Many published models still lack transparent reporting of data preprocessing, feature selection, hyperparameter tuning, missing-data handling, and model calibration. In addition, potential sources of bias may arise from imbalanced subtype distribution, under-representation of racial or ethnic groups, differences in treatment access, and heterogeneity in endpoint definitions (Chen et al., 2023). Future studies should therefore place greater emphasis on multicenter external validation, prospective testing, subgroup performance assessment, reproducible analytical pipelines, and reporting of model failure modes. These steps are essential for determining whether AI-enabled multi-omics models can generalize across institutions, populations, assay platforms, and real-world clinical workflows (Matta et al., 2024).

### 9.2 Modality-specific limitations and translational maturity

The translational maturity of AI-enabled multi-omics integration varies substantially across data modalities (Liu F. et al., 2026). Conventional imaging and radiomics are relatively scalable because standard clinical imaging is already widely available, but their reproducibility depends on imaging protocols, scanner type, reconstruction parameters, segmentation methods, and feature-extraction pipelines (El-Jammal and Chung, 2025). Digital pathology and pathomics provide rich information on tissue architecture, tumor morphology, stromal features, tumor-infiltrating lymphocytes, and biomarker-adjacent patterns, but their generalizability may be affected by staining variability, slide scanners, tissue processing, annotation strategies, and laboratory workflows (Datwani et al., 2025).

Bulk genomics and transcriptomics are more clinically established for selected assays and molecular classifiers, but broad AI-based multi-omics fusion remains less mature (El et al., 2025). High-dimensional molecular features combined with limited cohort sizes increase the risk of overfitting and unstable feature selection. Proteomics and metabolomics can provide information closer to functional tumor phenotypes, but they are often limited by high cost, preanalytical variability, platform-specific effects, and smaller validation cohorts (Creighton, 2024). Liquid biopsy offers minimally invasive and repeatable sampling for longitudinal monitoring, but AI-based liquid-biopsy models may be influenced by tumor-shedding heterogeneity, assay sensitivity, sampling time, and disease stage. Single-cell and spatial omics provide high-resolution information on cell states, spatial niches, tumor immune interactions, and treatment-resistance mechanisms, but they remain predominantly exploratory because of high cost, low throughput, analytical complexity, sparse data, and limited clinical standardization (Stylianakis et al., 2026).

The high cost of multi-omics testing, particularly spatial transcriptomics and deep metagenomics, has limited its broad adoption in routine breast cancer screening and recurrence monitoring (Moses and Pachter, 2022). Patients with advanced breast cancer may also require rapid treatment decisions before immunotherapy, whereas the turnaround time for sample collection, high-throughput sequencing, multimodal data cleaning, and generation of an AI-based prediction report may not meet clinical time constraints (Mateo et al., 2022). Balancing high-dimensional accuracy with clinical timeliness is therefore a practical requirement for implementation (Elemento et al., 2021).

From an implementation perspective, cost and turnaround time should be considered alongside model accuracy. Standardized sampling, streamlined sequencing workflows, automated quality control, and clinically interpretable reporting pipelines may help reduce delays, but these solutions require prospective evaluation before they can be incorporated into routine decision-making (Rajpurkar and Lungren, 2023; Bressan et al., 2023). Therefore, the clinical value of AI-driven multi-omics should be judged not only by predictive performance but also by feasibility, reproducibility, and timeliness (Elemento et al., 2021).

### 9.3 Clinical implementation, regulation, and governance

Liability, bias, and consent also require explicit planning. AI-assisted decisions may involve shared responsibility among clinicians, hospitals, software developers, laboratories, and device manufacturers, particularly when a model recommends treatment escalation or de-escalation, or biomarker-guided therapy selection (Mello and Guha, 2024). In practice, responsibility boundaries should be defined before deployment, including who reviews model outputs, who may override recommendations, how disagreements are documented, and how adverse decisions are audited. Bias across racial or ethnic, geographic, socioeconomic, and genetic-ancestry groups can arise from under-representation in genomic datasets, differences in screening access, local treatment patterns, and heterogeneity in clinical endpoint definitions (Viswanathan et al., 2024). Therefore, subgroup performance, calibration, uncertainty, and failure modes should be evaluated across clinically relevant populations before broad deployment. Data governance should therefore include federated or privacy-preserving learning where appropriate. It should also incorporate audit trails, data-use agreements, role-based access control, re-consent or broad consent for secondary multi-omics analysis, and patient-facing explanations of how samples, imaging data, molecular profiles and clinical records may be used to train or validate AI systems (Stark et al., 2025).

Regulatory and governance considerations are equally important. From the perspective of AI-based medical devices, regulatory agencies increasingly emphasize the intended use of the model, predefined change-control plans, risk management, human factors, and real-world performance monitoring (Health, 2025). These expectations are consistent with lifecycle oversight rather than one-time approval, because multi-omics models may drift when sequencing platforms, imaging protocols, tissue-processing workflows, patient ancestry, treatment patterns, or endpoint definitions change. Cybersecurity should also be addressed as part of medical-device software design, labeling, and premarket documentation (Health, 2026). The FDA framework for AI-enabled device software and predetermined change control plans highlights that AI-enabled devices intended to be modified over time require clear boundaries for future modification, while the EMA and broader European regulatory framework similarly stresses lifecycle oversight, data quality, transparency, accountability, and human oversight, together with a risk-based approach to model use. For breast-cancer multi-omics AI, these requirements imply that a model should have a locked clinical indication, documented training data provenance, prespecified update procedures, monitoring for algorithmic drift, periodic recalibration or revalidation when input distributions shift, post-deployment performance surveillance, audit trails for model outputs, and a mechanism for clinician override. Together, these safeguards are necessary to ensure that AI-enabled breast-cancer multi-omics models remain clinically auditable, ethically responsible, and aligned with real-world clinical workflows (Wang et al., 2023).

Omics data involves patient privacy, and the volume of data from a single center is often insufficient to train large deep learning models, federated learning enables collaborative model training through the exchange of model parameters without sharing raw data (Pati et al., 2022; Ogier du Terrail et al., 2023). The ProCanFDL project demonstrates the application of federated learning to proteomics data, successfully training a cancer subtype classifier that outperforms models from individual centers while protecting privacy. This is particularly important for collecting data on rare breast cancer subtypes (Cai et al., 2025). Figure 4 summarizes key challenges in the clinical application of AI-enabled multi-omics and potential strategies to address them.

**FIGURE 4:**
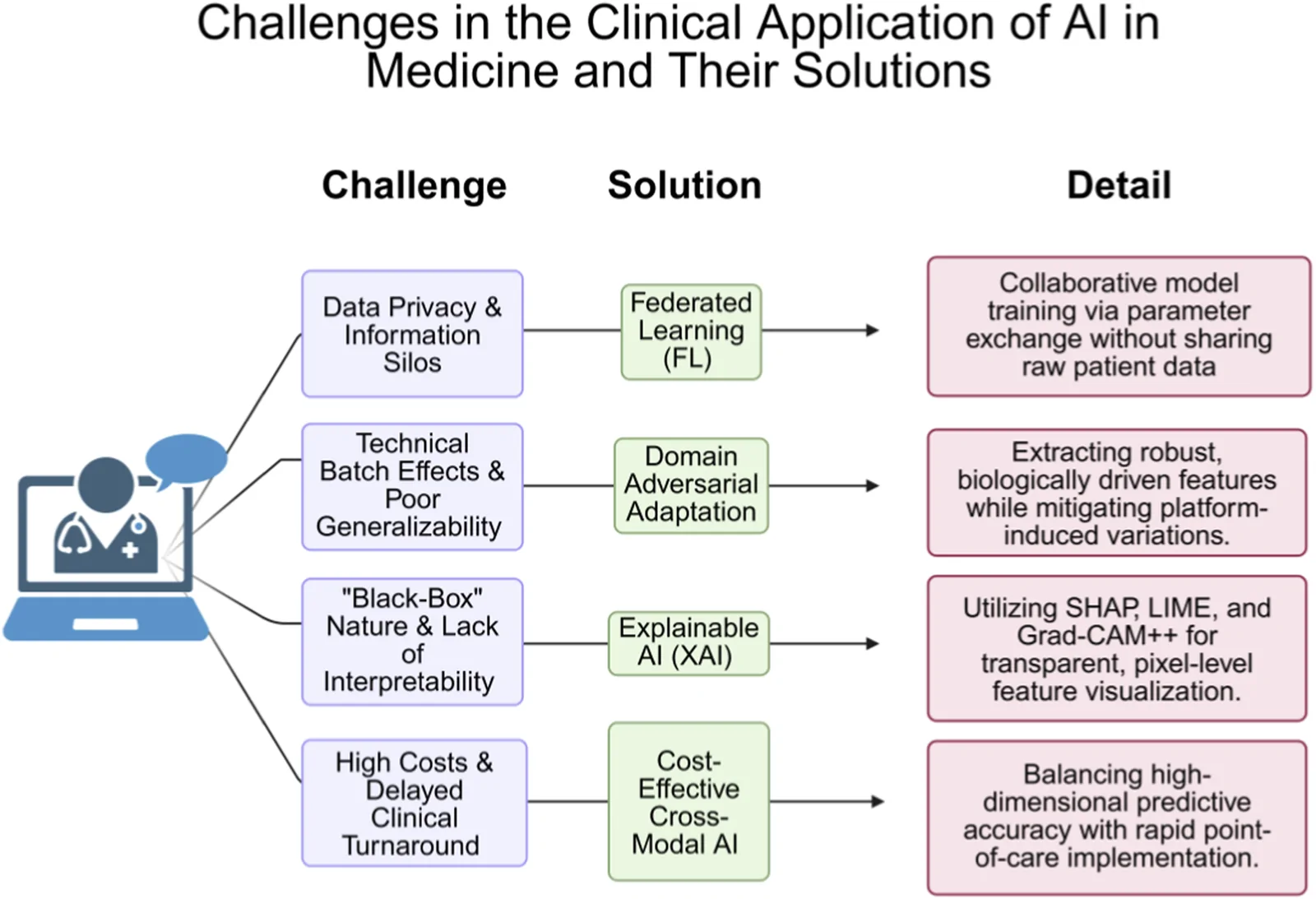
Challenges in the Clinical Application of AI in Medicine and Their Solutions. The figure illustrates the challenges currently facing the clinical application of AI and multi-omics, including data privacy and “data silos,” batch effects and generalization capabilities, “black-box” characteristics and lack of interpretability, as well as high costs and insufficient clinical timeliness. Potential solutions are listed below.

For complex pathological and high-dimensional imaging data, explainable AI (XAI) algorithms play an important role (Salahuddin et al., 2022). In a DNN-based study of early breast cancer detection that achieved 99.2% accuracy, high accuracy alone was insufficient to ensure clinician confidence. SHAP and lime were therefore incorporated to quantify the contribution of individual features to each prediction. For example, SHAP analysis indicated that the “concave points” feature had the strongest positive influence on malignancy prediction, translating abstract model weights into interpretable morphological guidance (Ghasemi et al., 2024).

### 9.4 Future directions for robust clinical translation

Future development of AI-enabled multi-omics integration in breast cancer should move from performance-driven model development toward clinically robust evidence generation (Akhoundova and Rubin, 2022). First, studies should prioritize larger and more diverse multicenter cohorts, with predefined clinical endpoints and adequate representation of molecular subtypes, disease stages, treatment settings, and demographic groups (Collins et al., 2024). Second, model development should include rigorous strategies to reduce overfitting, including appropriate separation of training, validation, and test sets; nested cross-validation where feasible; transparent feature selection; regularization; calibration assessment; and independent external validation (Andaur Navarro et al., 2021). Third, reproducibility should be improved through standardized reporting of preprocessing pipelines, model architectures, hyperparameters, software versions, feature definitions, and missing-data handling (Norgeot et al., 2020).

Fourth, future studies should evaluate clinical utility rather than relying only on discrimination metrics. Decision-curve analysis, calibration, subgroup analysis, prospective validation, workflow-impact assessment, cost-effectiveness analysis, and reporting of failure modes will be important for determining whether AI models can improve clinical decision-making. Fifth, privacy-preserving and collaborative learning strategies, including federated learning, may help overcome data-silo barriers while protecting patient privacy (Zhang F. et al., 2024). Finally, regulatory-grade AI models should be developed with locked intended use, transparent data provenance, lifecycle monitoring, mechanisms for clinician override, and post-deployment surveillance. These directions may help shift AI-enabled breast-cancer multi-omics integration from exploratory modeling toward clinically reliable decision support (Jiang et al., 2026).

## 10 Conclusion

This review summarizes progress in AI-driven multi-omics technologies for predicting immunotherapy response, elucidating resistance mechanisms, and assessing prognosis in breast cancer. It covers multimodal integration strategies involving radiomics, genomics, proteomics, spatial transcriptomics, and liquid biopsy, and discusses key barriers to clinical translation, including data privacy, batch effects, model interpretability, cost, and workflow integration.

This review contributes to the field by synthesizing evidence across four dimensions. First, it addresses an important area at the intersection of AI and breast cancer multi-omics, whereas many previous reviews have focused on single-modality AI applications or broader summaries of AI in oncology. Second, it highlights how multi-omics AI models may improve patient stratification and treatment-response prediction beyond single biomarkers such as PD-L1 expression or tumor-infiltrating lymphocytes, thereby helping to identify patients most likely to benefit from immunotherapy and reducing ineffective treatment. Third, it outlines potential translation strategies, including federated learning and domain-adaptive learning, which may mitigate privacy and generalizability barriers. Fourth, it identifies persistent gaps, including insufficient interpretability, limited prospective validation, and the absence of standardized multicenter datasets, which should be prioritized in future research.

In summary, AI-driven integration of multi-omics data provides a promising framework for breast cancer diagnosis, immunotherapy-response prediction, and prognostic assessment. By combining large-scale datasets with machine-learning and foundation-model approaches, AI may reduce some sources of human error and subjective variability and support more standardized diagnostic and prognostic workflows. In clinical practice, standardized operating procedures will be needed to clarify how AI models generate diagnoses, risk predictions, and treatment recommendations. Future work should strengthen translational research by incorporating larger and more diverse datasets, improving reproducibility, conducting prospective clinical trials, and establishing regulatory frameworks for ethical and safe AI use. These efforts will be essential for integrating AI into routine breast cancer care, improving patient outcomes, and reducing clinician workload. At the same time, the field should avoid premature claims of clinical readiness: most single-cell, spatial-omics, and high-dimensional fusion models remain discovery tools until they demonstrate reproducibility, calibration, cost-effectiveness, fairness, and benefit in prospective clinical workflows.

## Glossary

AI: Artificial Intelligence
AUC: Area Under the Curve
AUROC: Area Under the Receiver Operating Characteristic Curve
BRCA1/2: Breast Cancer Gene 1/2
CDK4/6: Cyclin-Dependent Kinase 4/6
CNNs: Convolutional Neural Networks
COL6A3: Collagen Type VI Alpha 3 Chain
CTCs: Circulating Tumor Cells
ctDNA: Circulating Tumor DNA
CXCL9: C-X-C Motif Chemokine Ligand 9
CXCR3: C-X-C Motif Chemokine Receptor 3
DFS: Disease-Free Survival
DNNs: Deep Neural Networks
ER: Estrogen Receptor
FAO: Fatty Acid Oxidation
FDG-PET/CT: Fluorodeoxyglucose Positron Emission Tomography/Computed Tomography
FISH: Fluorescence *In Situ* Hybridization
FSTL1: Follistatin-Like 1
GANs: Generative Adversarial Networks
GCNs: Graph Convolutional Networks
GDSC: Genomics of Drug Sensitivity in Cancer
GNNs: Graph Neural Networks
GraphSAGE: Graph Sample and Aggregate
H&E: Hematoxylin and Eosin
HER2: Human Epidermal Growth Factor Receptor 2
HIF-1α: Hypoxia-Inducible Factor 1-Alpha
HLA: Human Leukocyte Antigen
ICB: Immune Checkpoint Blockade
ICI: Immune Checkpoint Inhibitor
IHC: Immunohistochemistry
irAEs: Immune-Related Adverse Events
ITH: Intratumoral Heterogeneity
Ki-67: Ki-67 Proliferation Marker
KRAS: KRAS Proto-Oncogene, GTPase
LightGBM: Light Gradient Boosting Machine
LIME: Local Interpretable Model-Agnostic Explanations
LLMs: Large Language Models
LOH: Loss of Heterozygosity
LORIS: LOgistic Regression-Based Immunotherapy-Response Score
LR.score: Ligand–Receptor Score
METABRIC: Molecular Taxonomy of Breast Cancer International Consortium
MIL: Multiple Instance Learning
MIWAE: Missingness Imputation with Weighted Autoencoders
ML: Machine Learning
MOFS: Multi-Omics Feature Selection
MOGONET: Multi-Omics Graph cOnvolutional NETworks
MRI: Magnetic Resonance Imaging
mRNA: Messenger Ribonucleic Acid
MSFN: Multi-Omics Stacked Fusion Network
MSI: Mass Spectrometry Imaging
OPLS: Orthogonal Partial Least Squares
pCR: Pathological Complete Response
PCR: Polymerase Chain Reaction
PD-1: Programmed Cell Death Protein 1
PD-L1: Programmed Death-Ligand 1
PERCEPTION: PERsonalized Single-Cell Expression-Based Planning for Treatments In ONcology
PI3K: Phosphatidylinositol 3-Kinase
PIK3CA: Phosphatidylinositol-4,5-Bisphosphate 3-Kinase Catalytic Subunit Alpha
PR: Progesterone Receptor
PRSs: Polygenic Risk Scores
PSNs: Patient Similarity Networks
QALY: Quality-Adjusted Life-Year
RCB: Residual Cancer Burden
RFU: Repertoire Functional Unit
RNNs: Recurrent Neural Networks
RNA-seq: RNASequencing
scRNA-seq: Single-Cell RNA Sequencing
SHAP: SHapley Additive exPlanations
TCGA: The Cancer Genome Atlas
TCR: T-Cell Receptor
TILs: Tumor-Infiltrating Lymphocytes
TIME: Tumor Immune Microenvironment
TLS: Tertiary Lymphoid Structures
TMB: Tumor Mutational Burden
TME: Tumor Microenvironment
TNBC: Triple-Negative Breast Cancer
TNM: Tumor–Node–Metastasis
TRS: Thermalytix Risk Score
ViT: Vision Transformer
WES: Whole-Exome Sequencing
WSIs: Whole-Slide Images
XAI: Explainable Artificial Intelligence
